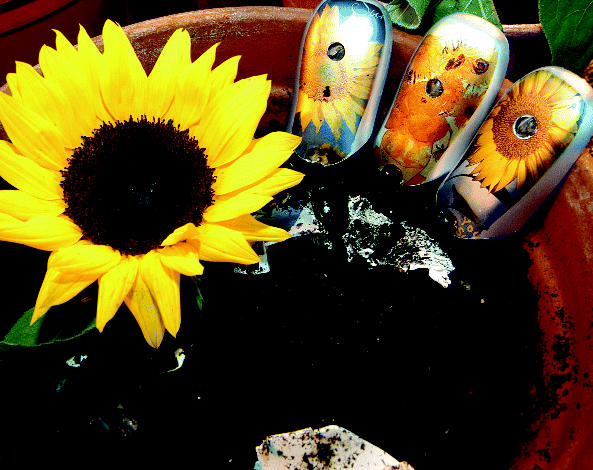# The Beat

**Published:** 2005-04

**Authors:** Erin E. Dooley

## Vive la Petite Auto!

Starting in January 2005 French drivers who buy large vehicles must pay a surcharge of up to about US$4,700 as part of a plan drawn up by the French Environment Ministry. The plan also rewards purchasers of smaller, cleaner vehicles with a rebate of up to US$935; the revenue from the sale of the 350,000 large vehicles typically sold each year is expected to pay for the small car rebates. This plan follows a yet-to-be-passed resolution to restrict SUVs in Paris’s city center and tourist areas. A recent study by the French government found that some 7% of premature deaths from lung cancer and cardiovascular and respiratory problems in France could be directly linked to vehicle emissions.

## China Goes Retro

In Beijing alone, nearly 1,000 new vehicles hit the road each day, and several Chinese metropolises (including Beijing) are among the world’s most polluted cities. Now a U.S.–Chinese partnership aims to turn the tide. In November 2004 the U.S. EPA, China’s State Environmental Protection Administration, the Beijing Environmental Protection Bureau, and other groups began a demonstration plan to retrofit an existing Chinese fleet of buses and trucks with clean diesel technology. The EPA has pledged $250,000 and a significant number of man-hours to the effort. Emissions from older diesel vehicles pose serious health problems and contribute to acid rain and ozone formation. Retrofitting should reduce emissions in the test diesel fleet by 40% or more.

## Green Journalism in Mozambique

The Mozambican Grupo Ambiental de Jornalistas, with assistance from the Blacksmith Institute, has begun producing and broadcasting environmental health programs on Radio Mozambique’s two main channels. Topics to date have included the causes and prevention of cholera (an intractable problem for the country for the past 15 years), the potential impact on crops and wildlife of the Cahora Bassa hydroelectric dam now under construction, benefits of unleaded gasoline (Mozambique is currently phasing out leaded gas), the need to conserve water, and tips for general sanitation. The group hopes to give the general public a sense of how their environment affects their health, and to promote discussion and environmental health advocacy.

## An End to Idle Threats?

Thanks to a new electrified system developed by IdleAire Technologies and currently used at 23 truck stops across the country, truckers can do their job in a cleaner way. Long-haul truckers are required to take a 10-hour rest stop for every 11 hours on the road. During that time, they traditionally keep their diesel engines running to power heaters, televisions, and other amenities inside the cab. Now they can plug into an IdleAire unit to meet a variety of electricity needs.

Idling trucks use about 1 billion gallons of fuel each year. The resulting diesel particles can cause a number of conditions including asthma, lung cancer, and heart disease. Installation of the IdleAire system at just one New Jersey truck stop eliminated 140 tons of pollution and saved 19,000 gallons of diesel fuel in its first six weeks of use.

## Defining the Sprawlscape

Yale urbanism and architecture professor Dolores Hayden has written *A Field Guide to Sprawl* to provide urban planners and sustainable-growth advocates with a common language for describing the sprawl landscape. The idea for the guide stemmed from Hayden’s difficulty in articulating to her students the new urban configurations often brought about by rapid and unchecked growth. The guide, released in July 2004, contains 51 novel “sprawl species” such as *toad* (short for “**t**emporary, **o**bsolete, **a**bandoned, or **d**erelict site”) and *snout houses* (homes characterized by prominent full-frontal garages). Hayden amassed the entries after searching industry websites, newspaper columns, real estate manuals, and planning glossaries.

## Phone-y Flowers

With 80 million new wireless phones sold in North America each year and the average user upgrading to a new phone every 18 months, the disposal of old phones is a huge problem. The phones not only take up valuable landfill space, but also contain toxic metals such as lead and cadmium that can leach into the environment. Researchers at Britain’s University of Warwick working with Motorola and the materials company PVAXX Research and Development have come up with a way to stem at least part of the problem in a unique way. They have developed a special polymer phone casing with a high-quality finish—and a twist: it’s compostable. For a final flourish, the designers embedded flower seeds in the casing, which germinate when the cover is composted.

## Figures and Tables

**Figure:**
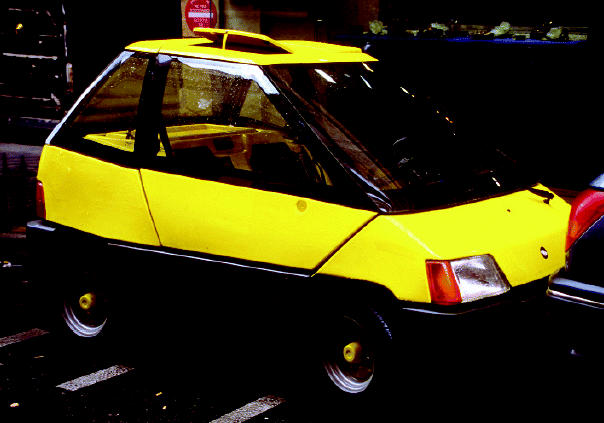


**Figure:**
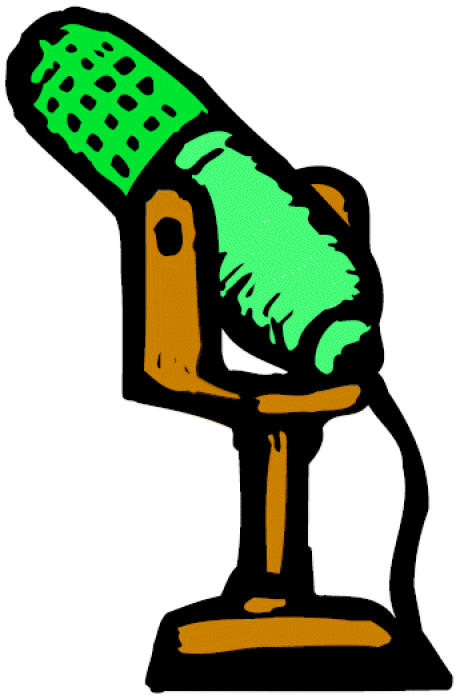


**Figure:**
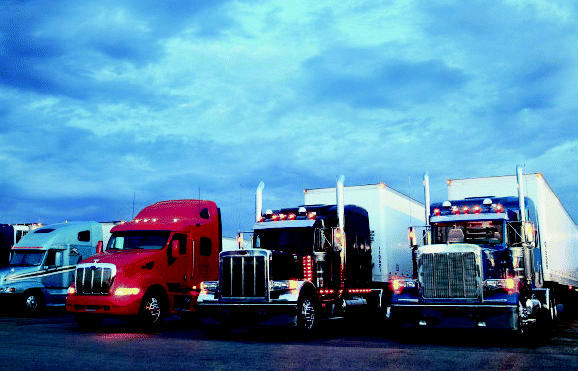


**Figure:**